# Ageing Study of Palm Oil and Coconut Oil in the Presence of Insulation Paper for Transformers Application

**DOI:** 10.3390/ma11040532

**Published:** 2018-03-30

**Authors:** Nur Aqilah Mohamad, Norhafiz Azis, Jasronita Jasni, Mohd Zainal Abidin Ab Kadir, Robiah Yunus, Zaini Yaakub

**Affiliations:** 1 Centre for Electromagnetic and Lightning Protection Research (CELP), Department of Electrical and Electronic Engineering, Faculty of Engineering, Universiti Putra Malaysia, 43400 Serdang, Selangor, Malaysia; qieylaqilah@gmail.com (N.A.M.); jasronita@upm.edu.my (J.J.); mzk@upm.edu.my (M.Z.A.A.K.); 2 Institute of Advanced Technology (ITMA), Universiti Putra Malaysia, 43400 Serdang, Selangor, Malaysia; 3 Institute of Power Engineering (IPE), Universiti Tenaga Nasional, 43000 Kajang, Selangor, Malaysia; 4 Department of Chemical and Environmental Engineering, Faculty of Engineering, Universiti Putra Malaysia, 43400 Serdang, Selangor, Malaysia; robiah@upm.edu.my; 5 Hyrax Oil Sdn. Bhd, Lot 4937 Batu 5 1/2, Jalan Meru, Mukim Kapar, 41050 Klang, Selangor, Malaysia; zaini@hyraxoil.com

**Keywords:** palm oil, coconut oil, insulation paper, thermal ageing, dielectric and physicochemical measurements

## Abstract

This paper presents a sealed ageing study of palm oil (PO) and coconut oil (CO) in the presence of insulation paper. The type of PO under study is refined, bleached, and deodorized palm oil (RBDPO) olein. Three different variations of RBDPO and one sample of CO are aged at temperatures of 90 °C, 110 °C, and 130 °C. The properties of RBDPO and CO as well as paper under ageing are then analysed through dielectric and physicochemical measurements. It is found that the effect of ageing is not significant on the alternating current (AC) breakdown voltages and relative permittivities of RBDPO and CO. There is a slight increment trend of the resistivity for CO, while for all of the RBDPO, the resistivity slightly decreases as the ageing progresses. Only CO shows an apparent reduction of the dielectric dissipation factor. Throughout the ageing time, the acidities of all of the RBDPO and CO remain at low level, while the moisture in oils decreases. The tensile index (TI) of the papers for all of the RBDPO and CO retain more than 50% of the TI. A significant increment of the paper ageing rates of all of the RBDPO and CO is observed at an ageing temperature of 130 °C.

## 1. Introduction

Vegetable oils are among the viable alternatives of dielectric insulation fluids that have been identified for application in transformers. In recent years, a number of laboratory studies have been carried out to examine the suitability of these oils; these covered different aspects, including their dielectric, physical, and chemical properties [1,2,3,4]. Vegetable oils have also been tested and applied in transformers at different voltage levels [5].

To date, different types of vegetable oils have been proposed for potential application in transformers usage, such as rapeseed, sunflower, olive, castor, palm and coconut oils [1,2,4,6,7,8,9,10,11,12]. Several studies have also been carried out on chemically modified vegetable oils [13,14,15,16]. Palm oil (PO) and coconut oil (CO) are among the common vegetables oils available in Asian countries [17,18,19]. Crude palm oil, palm kernel oil and refined, bleached, and deodorized palm oil (RBDPO) are the types of PO that can be extracted from the palm nut [20]. The fractionation process of RBDPO will result in RBDPO olein [21]. CO is obtained from the kernel of matured coconut palms, which can be further processed to produce virgin and refined coconut oil [22,23].

Since the failure of in-service transformers could be costly, it is essential to understand the oil characteristics; this can be carried out through accelerated thermal ageing study. Knowledge is well established on the ageing characteristics of mineral oil (MO), as it has been studied extensively [24,25,26,27]. On the other hand, the ageing studies on vegetable oils are currently ongoing [1,3,13,25,27,28,29,30,31,32,33,34,35,36,37,38,39]. These studies showed that the vegetable oils had slight different ageing characteristics than conventional MO. A few studies found that vegetable oils could provide a protective mechanism for insulation paper during ageing [35,37,38]. It was suggested that the ageing of paper in vegetable oils could be delayed due to hydrolytic protection and water-scavenging mechanisms [35,37,38]. Several studies on different types of vegetable oils found that most of their electrical properties were not significantly affected by ageing [3,33,36]. Wilhelm et al. and Ciuriuc et al. showed that ageing affected only the dielectric dissipation factor and resistivity [33,36]. One of the studies found that there was a clear reduction trend of resistivity for vegetable oils in the presence of copper, iron, and insulation paper [31]. Another study found that as ageing progressed, the dielectric dissipation factor of vegetable oil could increase due to the presence of moisture in paper [33]. Ageing could also affect the physicochemical properties of vegetable oils [32,33,34,39]. One of the studies found that the viscosity and acidity of vegetable oils could significantly increase in the presence of oxygen [33,39]. The acidity of vegetable oils could also increase due to the presence of high moisture in insulation paper [33].

To date, the research on RBDPO has mostly focussed on the examination of its basic properties such as AC breakdown voltage, dielectric properties, viscosity, moisture, and acidity [40,41,42,43]. Several studies also examine the partial discharge characteristics of RBDPO, as well as the effect of moisture and fat content on the breakdown voltage, dielectric dissipation factor, viscosity, and flash point of RBDPO [44,45,46,47]. A previous ageing study on RBDPO was carried out under open conditions and without the presence of insulation paper [48]. On the other hand, a few thermal ageing studies have been carried out on CO that focussed on the oil properties such as interfacial tension, acidity, AC breakdown voltage and dielectric properties [49,50]. Other studies cover on the conductivity, frequency dielectric spectroscopy analysis, and gassing characteristics of CO under thermal/electrical faults [11,51]. There have also been a few studies on the lightning breakdown voltages of both RBDPO and CO [51,52,53,54]. This paper will focus not only on the general ageing characteristics of PO and CO; it will also focus on the mechanical strength performance of the transformer’s insulation paper in the presence of moisture. The type of PO under test is RBDPO olein. The dielectric and physicochemical properties RBDPO and CO, as well as the mechanical strength and moisture of the insulation paper, are measured. The Weidmann model was used to analyse the degradation rates of papers aged in RBDPO and CO.

## 2. Materials and Methods

### 2.1. Fluids under Test

A total of three samples of RBDPO olein and one sample of CO were investigated in this study. All of the RBDPO had different fat and vitamin E/A contents, which were obtained from the manufacturer’s datasheet, as shown in Table 1. All of the RBDPO had almost equal saturated and unsaturated fats contents, where only RBDPOA had vitamin A, and RBDPOC had the highest content of vitamin E. There was no vitamin E and A in CO, which mainly consisted of saturated fat. Further tests through the gas chromatography technique were conducted in order to determine the content of fatty acids in all of the RBDPO and CO according to the ISO 5508:1990, which can be seen in Table 1 [55]. The highest content of fatty acids in all of the RBDPO was oleic acid (monounsaturated), with percentages ranging between 41.1–43%, followed by palmitic acid (saturated), with percentages from 37.7% to 39.3%. CO is dominated by lauric and myristic acids (saturated), with percentages of 48.6% and 20%, respectively.

### 2.2. Thermal Ageing Procedure

First, the pre-processing procedures of RBDPO and CO were carried out by filtering the oils three times through a membrane filter with a pore size of 0.2 µm. Next, these oils were dried in a vacuum oven for two days at 85 °C, where the final moisture content of RBDPOA, RBDPOB, RBDPOC, and CO were 106 ppm, 126 ppm, 119 ppm, and 151 ppm respectively. The type of insulation paper used in this study was Kraft paper, which consists mainly of cellulose [56]. The paper was first dried in a vacuum oven at 105 °C with a pressure of 0.09 MPa for two days. The moisture content of the insulation paper after drying was around 2.8%. Next, the paper was impregnated with oil in a vacuum oven at 85 °C for 24 h. After the oil impregnation process, there was an increment of moisture in the oil, where the values for RBDPOA, RBDPOB, and RBDPOC were 330 ppm, 324 ppm, and 294 ppm, respectively, while the value for CO was 486 ppm. A decrement of moisture in paper was observed where the values for RBDPO were between 1.9–2.2%, while for CO, it was 1.2%. This phenomenon was expected, since the temperature was applied during the impregnation process, which could cause the migration of moisture from paper to oil. It is known that temperature can affect the migration of moisture from paper to oil [57]. The ratio of oil and paper used in this study was 20:1 (450 g oil and 22.5 g insulation paper). The ageing of oil and paper was carried out in a borosilicate glass, and in total, 30 samples of RBDPO and 10 samples of CO were aged with paper. In order to minimise contact with oxygen, all of the samples were filled with nitrogen. Bottle caps enforced with polytetrafluoroethylene (PTFE) tape were used to seal all of the samples in order to minimise the contact with the environment. The ageing temperatures were set at 90 °C, 110 °C, and 130 °C, while the ageing times were fixed to 0 days, 7 days, 14 days, and 28 days.

### 2.3. Test Description

#### 2.3.1. AC Breakdown Voltage

An automatic oil breakdown tester, BAUR DPA 75C, was used to carry out the AC breakdown measurement based on ASTM D 1816 at ambient temperature ranging between 28.4–32.9 °C [58]. The gap distance between two VDE electrodes, each with a diameter of 36 mm, was set to 1 mm. The voltage ramping rate was set to 0.5 kV/s, and a 5-min time interval was given between each breakdown. For each sample, 400 mL of oil was used for the AC breakdown test. An average value of 50 measurements of AC breakdown voltage was used in this study.

#### 2.3.2. Dielectric Dissipation Factor, Relative Permittivity and Resistivity

A BAUR DTL C oil tester was used to measure the dielectric dissipation factor, relative permittivity, and resistivity of the oils based on IEC 60247 [59]. For each sample, 40 mL of oil was tested at 90 °C. For this study, one measurement of dielectric dissipation factor, relative permittivity, and resistivity was taken for each RBDPO and CO.

#### 2.3.3. Viscosity

An automatic SVM 3000 Stabinger viscometer was used to determine the viscosity of the oils based on ASTM D 445 [60]. For each sample, 5 mL of oil was measured at 40 °C. For this study, one measurement of viscosity was taken for each RBDPO and CO.

#### 2.3.4. Moisture in Oil and Paper

A Metrohm 831 Karl Fischer (KF) Coulometer was used to measure the moisture in oil based on ASTM D 6304 [61]. For each sample, 1 mL of oil was used for the moisture in the oil measurement. The moisture content of the insulation paper was measured by a Metrohm 774 Karl Fischer Coulometer, according to IEC 60814 [62]. The moisture in the paper was extracted via an oven. In total, two measurements of moisture were taken for RBDPO, CO, and the insulation paper, where an average value was used for study.

#### 2.3.5. Acidity

A Metrohm 877 oil Titrano plus was used for the measurement of the acidity of the oils where the test was carried out based on ASTM D 974 [63]. For each sample, 10 g of oil was used for the acidity measurement. For this study, one measurement of acidity was taken for each RBDPO and CO.

#### 2.3.6. Tensile Index

Tensile strength (TS) is defined as the maximum tensile force per unit width that a paper and board can withstand before breaking [64,65]. The TS of paper is normally represented by the tensile index (TI). 

The TI measurement of paper was carried out by a 5 kN Universal Testing Machine, according to BS EN ISO 1924-2 [65]. The crosshead speed and full-scale load range were set to 20 mm/min and 0.5 kN [65]. The gap distance and width of the samples used for the tests were 180 mm ± 1 mm and 16 mm ± 0.1 mm. Once the maximum load was obtained, the TI of the paper was determined using Equation (1). In total, 10 measurements of TI were taken for each paper aged in RBDPO and CO, and an average value was used for study.

(1) $$TI=((\bar{F}/w)/G)\times 10^{3}$$

where TI is the tensile index of the paper in Newtons metres per grams, $\bar{F}$ is the maximum load in newtons, w is the width of the paper in millimetres, and G is the grammage of the paper in grams per square metre [65,66]. The grammage was determined based on mass per unit area of the paper, and in this study, the value was around 51.8 g/m^2^.

## 3. Result

### 3.1. Electric Properties of Oils

#### 3.1.1. AC Breakdown Voltage

**Figure 1:**
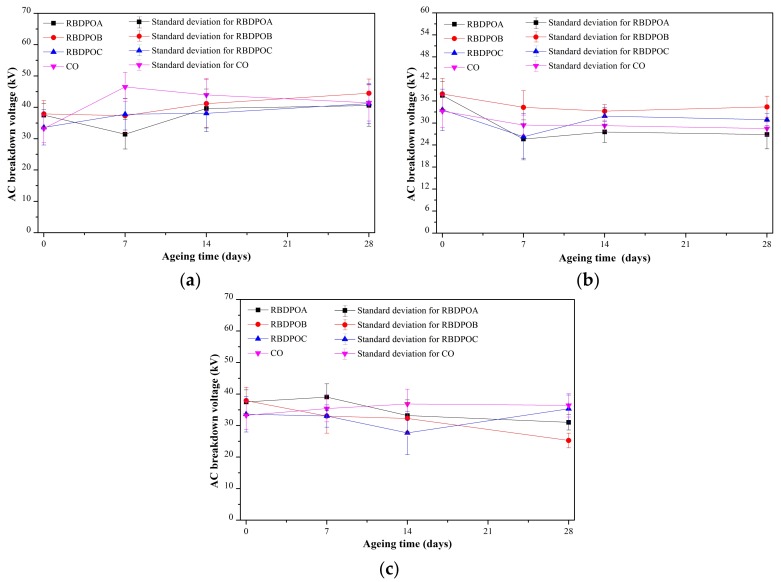
Variation of AC breakdown voltage for RBDPOA, RBDPOB, RBDPOC, and CO with ageing times at (**a**) 90 °C; (**b**) 110 °C and (**c**) 130 °C.

All of the samples showed no significant reductions of AC breakdown voltages. At an ageing temperature of 90 °C, there were slight increment trends of AC breakdown voltages for all of the RBDPO, as shown in Figure 1a. A slight reduction trend of AC breakdown voltage of CO was found after seven days of ageing.

The AC breakdown voltages of all of the RDBPO and CO initially decreased at an ageing temperature of 110 °C, as shown in Figure 1b. After seven days of ageing, RBDPOA, RBDPOB, and CO showed no significant changes in the AC breakdown voltages. On the other hand, the AC breakdown voltage of RBDPOC increased after seven days of ageing, and later slightly decreased after 14 days of ageing.

At an ageing temperature of 130 °C, only CO showed a slight increment trend of AC breakdown voltage, as shown in Figure 1c. After seven and 14 days of ageing, the AC breakdown voltages of RBDPOA and RBDPOB started to decrease. Meanwhile, the AC breakdown voltage of RBDPOC fluctuated between 27.7–35.3 kV.

#### 3.1.2. Dielectric Dissipation Factor

**Figure 2:**
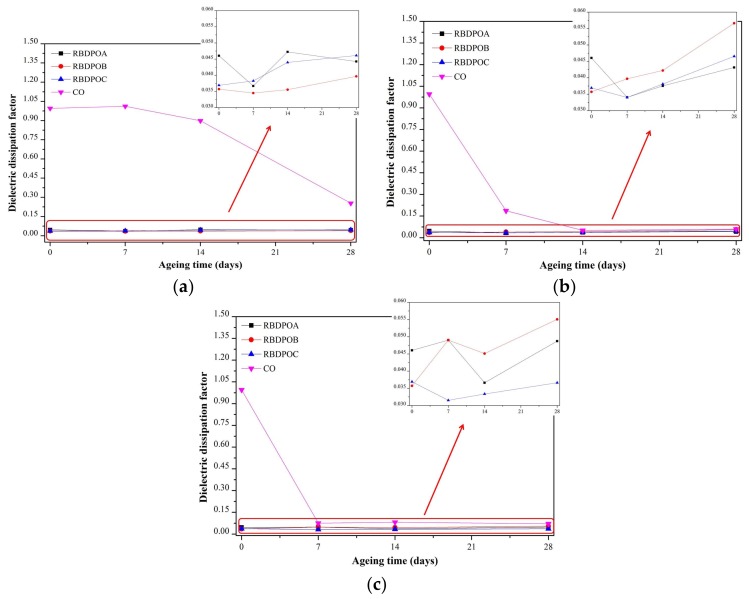
Variations in the dielectric dissipation factors for RBDPOA, RBDPOB, RBDPOC, and CO with ageing times at (**a**) 90 °C; (**b**) 110 °C; and (**c**) 130 °C.

All of the RBDPO had low dielectric dissipation factors ranging from 0.031 to 0.056 at all of the ageing temperatures. At an ageing temperature of 90 °C, the dielectric dissipation factor of CO started to show a significant reduction after seven days of ageing, whereas the value at the end of the ageing time was 0.25, as shown in Figure 2a.

At an ageing temperature of 110 °C, the dielectric dissipation factor CO suffered a 81.2% reduction during the initial stage of ageing, as shown in Figure 2b. The final value was 0.06 at the end of the ageing time.

The reduction pattern of the dielectric dissipation factor of CO at an ageing temperature of 130 °C was similar to that at an ageing temperature of 110 °C, as shown in Figure 2c. The dielectric dissipation factor of CO maintained at values between 0.071–0.082 from seven until 28 days of ageing.

#### 3.1.3. Relative Permittivity

**Figure 3:**
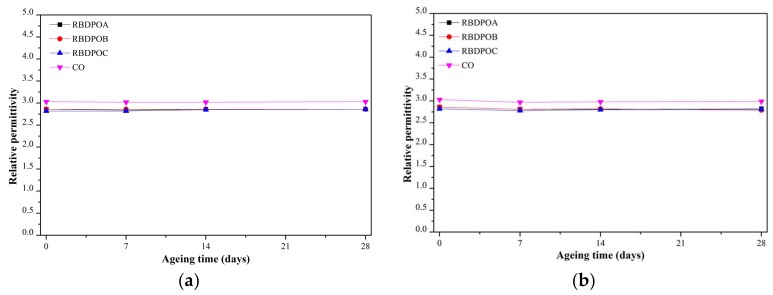

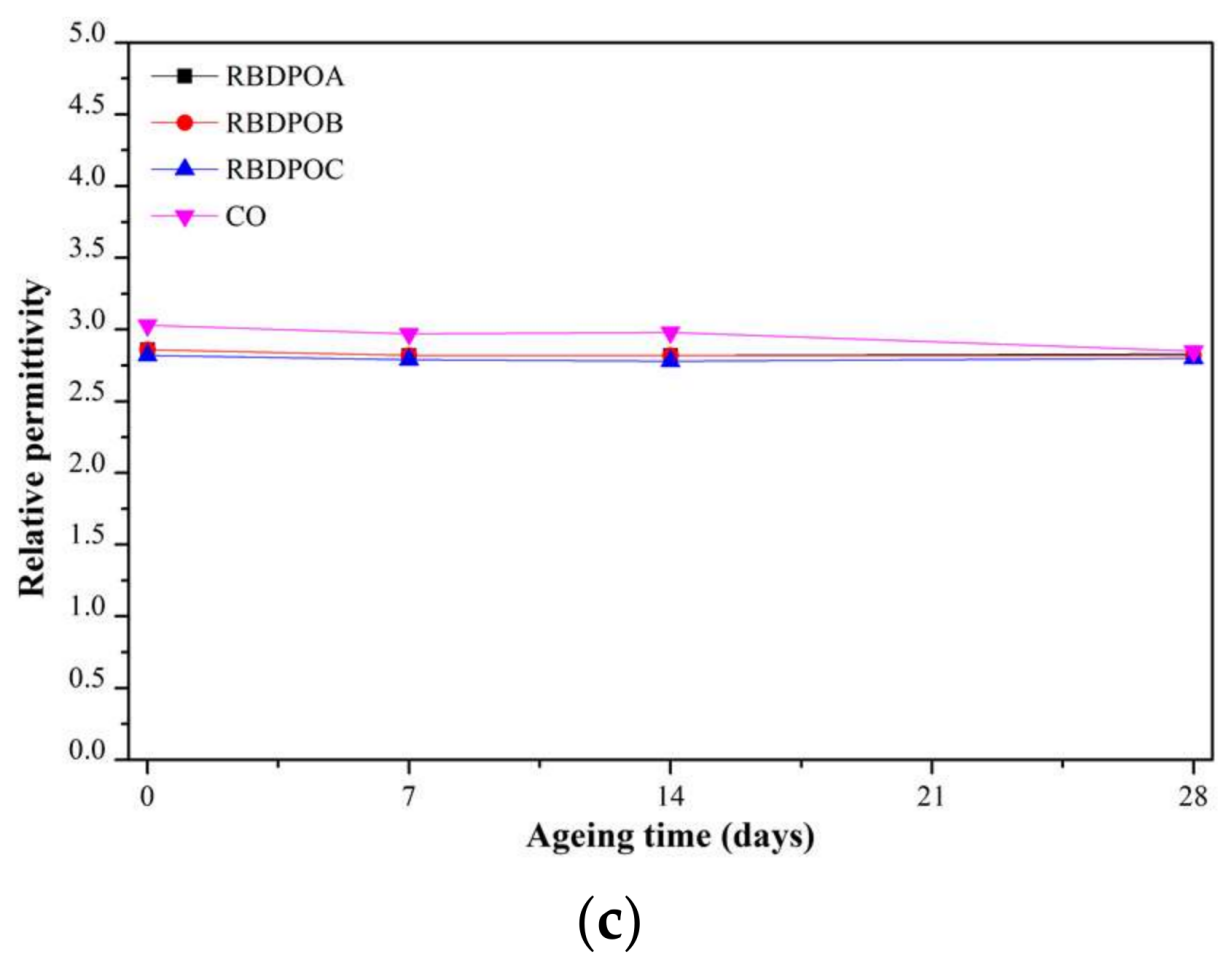
Variations in relative permittivity for RBDPOA, RBDPOB, RBDPOC, and CO with ageing time at (**a**) 90 °C; (**b**) 110 °C; and (**c**) 130 °C.

The relative permittivities of all of the RBDPO and CO were not affected by ageing, as shown in Figure 3a–c. Throughout the ageing time and at all of the ageing temperatures, the relative permittivities of all of the RBDPO maintained at values between 2.78–2.86. Meanwhile, the relative permittivity CO maintained at values between 2.85–3.03.

#### 3.1.4. Resistivity

**Figure 4:**
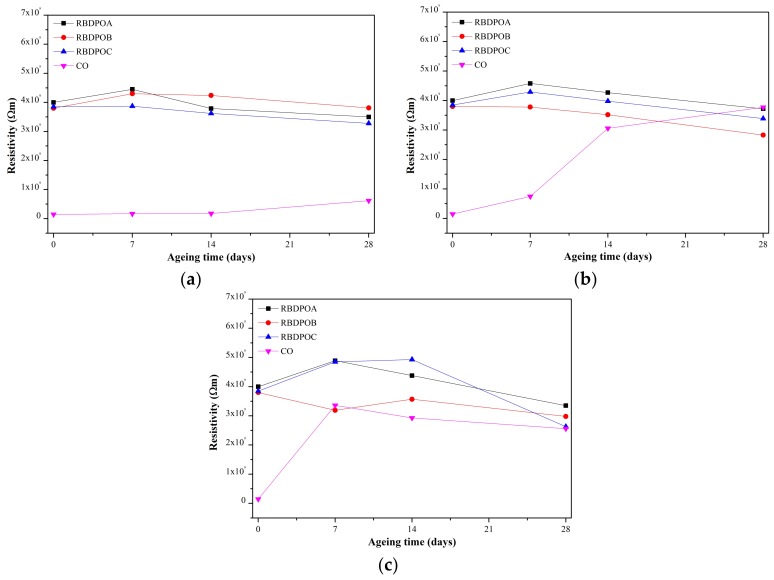
Variations of resistivity for RBDPOA, RBDPOB, RBDPOC, and CO with ageing time at (**a**) 90 °C; (**b**) 110 °C; and (**c**) 130 °C.

The resistivities for RBDPOA and RBDPOB initially increased, and started to show slight reduction trends after seven days of ageing at an ageing temperature of 90 °C, as seen in Figure 4a. Only RBDPOC showed a slight reduction trend of resistivity throughout the ageing time. On the other hand, the increment trend of resistivity for CO was apparent after 14 days of ageing, where the value after 28 days of ageing was 6.2 × 10^8^ Ωm.

At an ageing temperature of 110 °C, the resistivities for all of the RBDPO started to show a clear reduction trend from seven until 28 days of ageing, as seen in Figure 4b. There was a clear increment trend of CO throughout the ageing time.

The resistivities of RBDPOA and RBDPOC at an ageing temperature of 130 °C initially increased, and then started to decrease after seven and 14 days, respectively, as shown in Figure 4c. RBDPOB showed a slight reduction trend of resistivity throughout the ageing time. The resistivity of CO initially increased, and then started to slightly decrease after seven days of ageing.

### 3.2. Physicochemical Properties of Oils and Paper

#### 3.2.1. Viscosity

**Figure 5:**
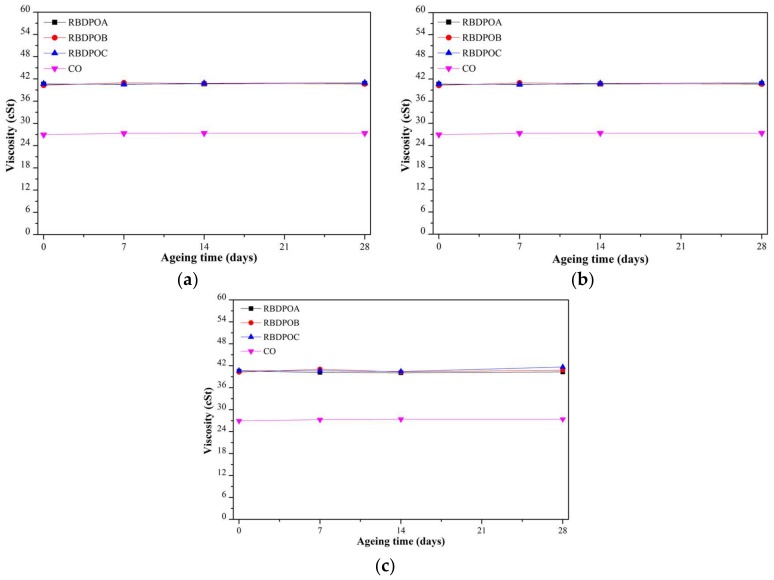
Variations of viscosity for RBDPOA, RBDPOB, RBDPOC, and CO with ageing time at (**a**) 90 °C; (**b**) 110 °C; and (**c**) 130 °C.

The viscosities of all of the samples remained almost unchanged at all of the ageing temperatures, as shown in Figure 5a–c. Throughout the ageing time, the range of the highest viscosities for all of the RBDPO was between 40.1 cSt and 41.7 cSt. On the other hand, the range of viscosity for CO was between 27.3–28.8 cSt.

#### 3.2.2. Moisture in Oil

**Figure 6:**
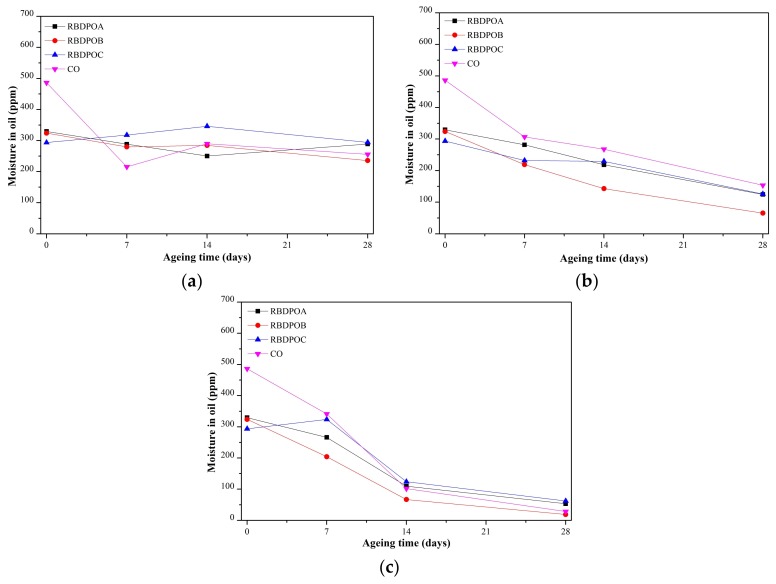
Variations of moisture in oil for RBDPOA, RBDPOB, RBDPOC, and CO with ageing time at (**a**) 90 °C; (**b**) 110 °C; and (**c**) 130 °C.

At an ageing temperature of 90 °C, the moisture in all of the RBDPO and CO showed no clear trends, as shown in Figure 6a. The moisture in CO initially decreased and fluctuated at values between 215–290 ppm after seven days of ageing.

At an ageing temperature of 110 °C, the moisture in all of the RBDPO and CO showed clear reduction trends, as shown in Figure 6b. At the end of the ageing time, the final moisture contents of all of the RBDPO were between 65–126 ppm, while the moisture content in CO was 153 ppm.

The moisture in oil for all of the RBDPO and CO at an ageing temperature of 130 °C showed similar reduction trends to that at an ageing temperature of 110 °C, as shown in Figure 6c. Initially, the moisture in all of the RBDPO and CO decreased significantly, and started to decrease at a slower rate after 14 days of ageing.

#### 3.2.3. Moisture in Paper

**Figure 7:**
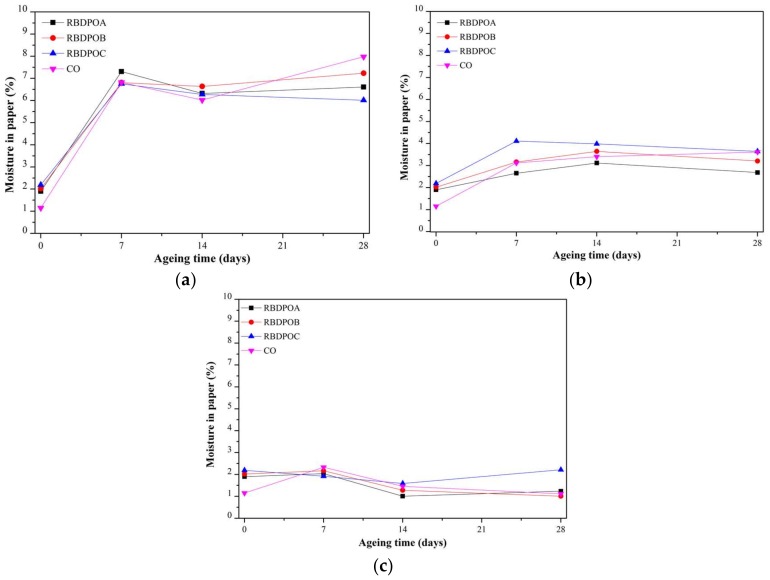
Variations of moisture in the paper for RBDPOA, RBDPOB, RBDPOC, and CO with ageing time at (**a**) 90 °C; (**b**) 110 °C; and (**c**) 130 °C.

At an ageing temperature of 90 °C, the moisture in paper for all of the RBDPO and CO increased significantly at the initial stage of ageing, as seen in Figure 7a. The moisture in paper for RBDPOA, RBDPOB, and CO slightly decreased after seven days of ageing, and increased after 14 days of ageing. The moisture in paper for RBDPOB continued to slightly decrease from seven days until the end of the ageing time.

At an ageing temperature of 110 °C, the moisture in the paper for all of the samples showed no clear patterns, as shown in Figure 7b. Initially, the moisture in the paper for most of the RBDPO slightly increased, and then started to slightly decrease after 14 days of ageing. On the other hand, the moisture in the paper for CO showed a slight increment trend throughout the ageing time.

The moisture in the paper for RBDPOA, RBDPOB, and CO at an ageing temperature of 130 °C slightly increased at the start of ageing, and later decreased after seven days of ageing, as shown in Figure 7c. On the other hand, the moisture in the paper for RBDPOC decreased from the start of ageing until 14 days of ageing. The moisture in the paper for RBDPOA and RBDPOC increased after 14 days of ageing, where the values at the end of the ageing time were 1.2% and 2.2%, respectively.

#### 3.2.4. Acidity

**Figure 8:**
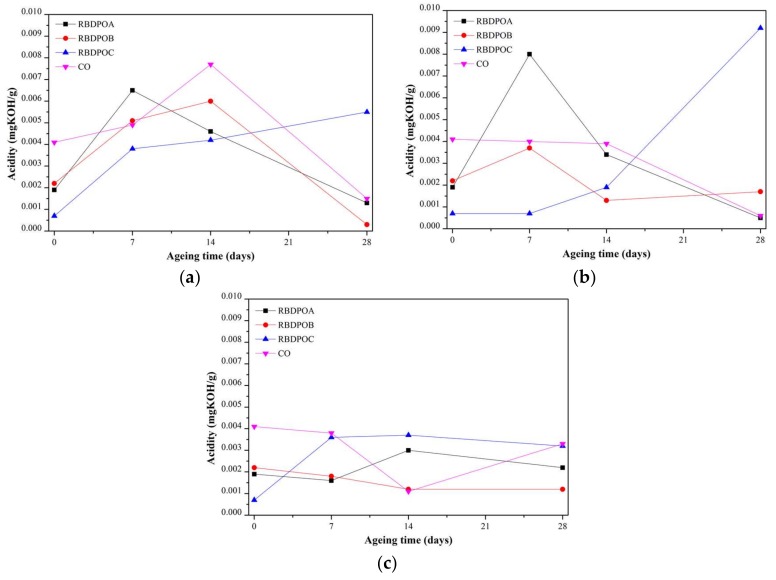
Variations of acidity for RBDPOA, RBDPOB, RBDPOC, and CO with ageing time at (**a**) 90 °C; (**b**) 110 °C; and (**c**) 130 °C.

At all of the ageing temperatures, all of the samples showed no clear trends of acidities, as shown in Figure 8. Throughout the ageing time, all of the RBDPO and CO had low acidities and maintained less than 0.01 mg KOH/g. The acidities of RBDPOB and CO at an ageing temperature of 90 °C initially increased, and then started to decrease after 14 days of ageing. After 28 days of ageing, the acidities of RBDPOB and CO were 0.0003 mg KOH/g and 0.0015 mg KOH/g, respectively, as shown in Figure 8a. On the other hand, the acidity of RBDPOA decreased almost linearly from seven days to 28 days of ageing, while RBDPOC showed a steady increment trend of acidity throughout the ageing time.

At an ageing temperature of 110 °C, the acidity for RBDPOC showed a clear increment trend, as shown in Figure 8b. RBDPOA, RBDPOB, and CO all experienced a reduction of acidities after seven days and 14 days of ageing, respectively.

At an ageing temperature of 130 °C, only RBDPOB showed a slight decrement of acidity, as shown in Figure 8c. Initially, the acidity of RBDPOC increased and then started to slightly decrease at the later stage of the ageing. The acidities of RBDPOA and CO showed no clear trends, and fluctuated between 0.0011–0.0041 mg KOH/g.

### 3.3. Tensile Strength of Insulation Paper

#### 3.3.1. Tensile Index (TI)

**Figure 9:**
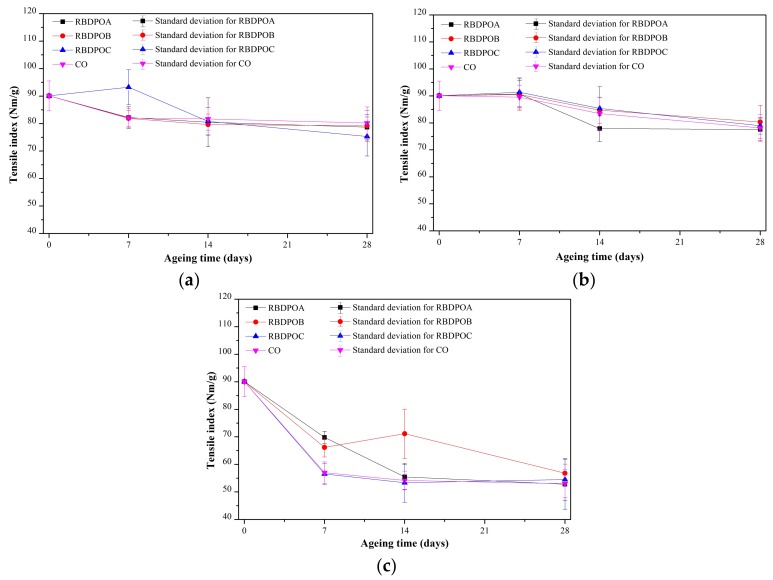
Variation of tensile indexes (Tis) for RBDPOA, RBDPOB, RBDPOC, and CO with ageing time at (**a**) 90 °C; (**b**) 110 °C; and (**c**) 130 °C.

At all of the ageing temperatures, the strength of the papers aged in all of the RBDPO and CO remained higher than the suggested criteria of 50% and 25% retentions of TI in IEEE standard C57.91-1995 [67]. At an ageing temperature of 90 °C, the papers aged in RBDPOA, RBDPOB, and CO experienced reductions of TI initially, and then maintained almost unchanged after seven days of ageing, as shown in Figure 9a. The paper aged in the RBDPOC experienced a reduction of TI after seven days of ageing, and continued to decrease until the end of the ageing time. At the end of the ageing time, all of the samples showed between 83.6–89.1% retention of TI.

At an ageing temperature of 110 °C, the papers for all of the samples experienced reductions of TI after seven days of ageing, as seen in Figure 9b. The percentage retention of TI for all of the samples was between 62–68.5% at the end of the ageing time.

At an ageing temperature of 130 °C, the papers of all of the samples showed clear TI reduction trends, as shown in Figure 9c. Initially, the papers for all of the samples experienced a significant reduction in TI. Only the TI of the paper aged in RBDPOA continued to decrease until the end of the ageing time, while there were no significant changes in the TI of the paper aged in either RBDPOC or CO after seven days of ageing. At the end of the ageing time, all of the samples showed a retention of TI between 58.6–63.1%.

#### 3.3.2. Rate of Paper Ageing Based on the Weidmann Model

The Weidmann model was used in this study to represent the relationship between TI and ageing time. The purpose of using this model was to obtain the degradation rate of papers aged in all RBDPO and CO, which can be determined by Equation (2) [68]. The Weidmann model only requires a few numbers of data, and this model provides a quick empirical solution to fit the measured TI data [69].

TI = TI_o_ exp ^−C^_TI_^t^ (2)

where TI_o_ is the initial tensile index of paper in Newtons metres per gram, C_TI_ is the ageing rate in days^−1^, and t is the ageing time in days.

**Figure 10:**
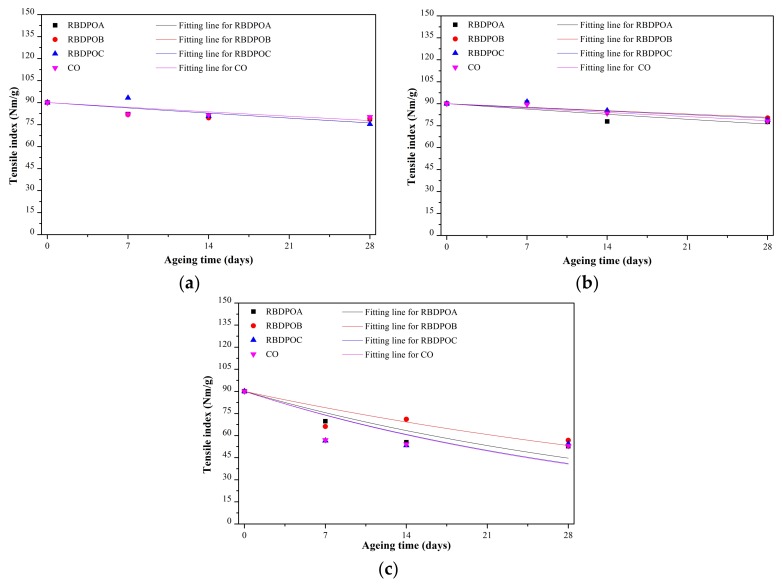
Fitting based on the Weidmann model for the tensile indexes of paper aged in RBDPOA, RBDPOB, RBDPOC, and CO at (**a**) 90 °C; (**b**) 110 °C; and (**c**) 130 °C.

The fittings of the TI of paper of all of the samples by the Weidmann model can be seen in Figure 10a–c. The paper ageing rates and confidence bounds of all of the samples at all of the ageing temperatures are shown in Table 2. At an ageing temperature of 90 °C, the paper ageing rates among the RBDPO were quite close, ranging between 0.597 × 10^−2^ and 0.6 × 10^−2^, while the paper ageing rate of CO was 0.523 × 10^−2^. At an ageing temperature of 110 °C, the paper ageing rate of RBDPOA was the highest, followed by RBDPOC and RBDPOB. The paper ageing rate of CO was 0.492 × 10^−2^. At an ageing temperature of 130 °C, the paper ageing rates of all of the RBDPO and CO were higher than at ageing temperatures of 90 °C and 110 °C. The paper ageing rates of RBDPOA, RBDPOB, RBDPOC, and CO at an ageing temperature of 130 °C were 4.17, 4.84, 6.76, and 5.78 times higher than the rates at an ageing temperature of 110 °C. 

## 4. Discussion

**Figure 11:**
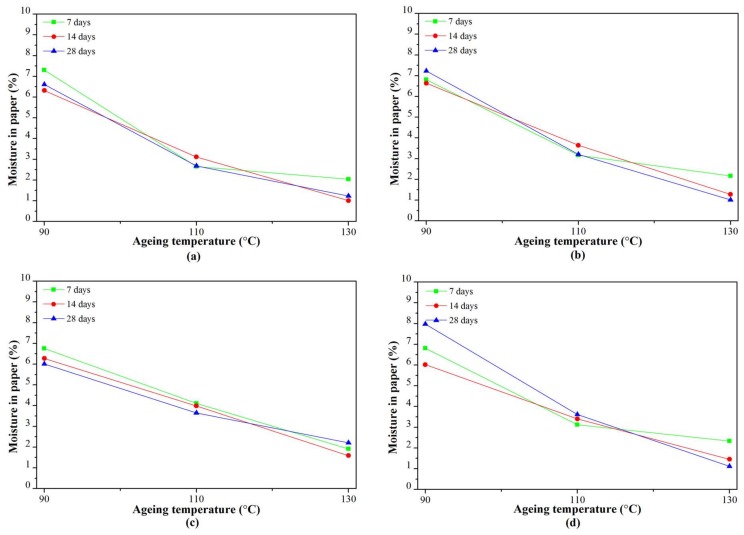
Variation of moisture in paper at seven days, 14 days, and 28 days with ageing temperature for (**a**) RBDPOA; (**b**) RBDPOB, (**c**) RBDPOC; and (**d**) CO.

**Figure 12:**
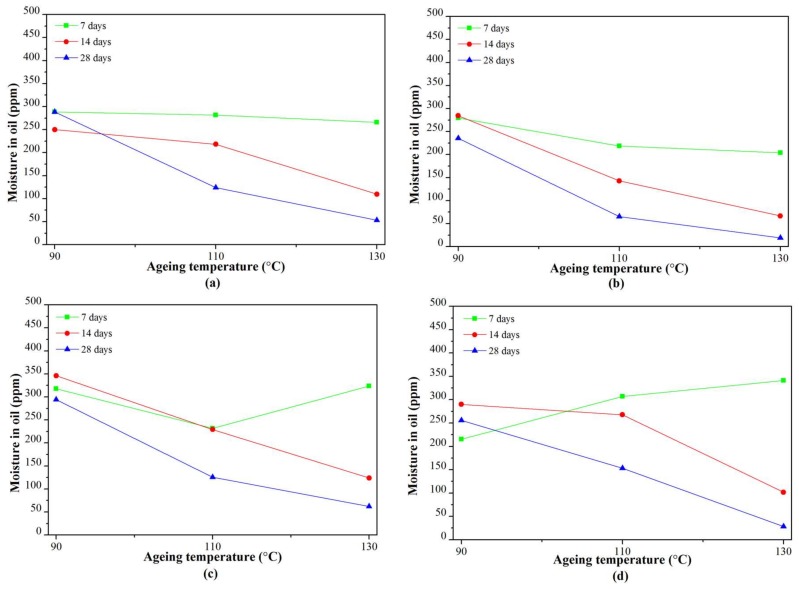
Variation of moisture in oil at seven days, 14 days, and 28 days with ageing temperature for (**a**) RBDPOA; (**b**) RBDPOB; (**c**) RBDPOC; and (**d**) CO.

Figure 11 is the representation of Figure 7 that shows the relationship between the moisture in the paper and the ageing temperature at different ageing times. The moisture in the paper for all of the RBDPO and CO decreased as the ageing temperature increased, which may indicate the migration of moisture from the paper to the oil. This phenomenon is also influenced by the ability of natural ester to absorb moisture [70]. Both RBDPO and CO are within a natural ester group that consists of triglyceride, which is made of glycerol and fatty acids [57]. However, natural ester is also able to undergo a hydrolysis process and consume the moisture, which could be the reason why the moisture in all of the RBDPO and CO decreased as the temperature increased, especially at 14 and 28 days of ageing, as shown in Figure 12. The reduction trends of moisture in all of the RBDPO and CO, especially at ageing temperatures of 110 °C and 130 °C, as shown in Figure 6, are also in line with previous studies [70]. The moisture reduction by the natural ester hydrolysis mechanism is also one of the possible reasons why there are no significant reductions of AC breakdown voltages for all of the RBDPO and CO throughout the ageing times, as shown in Figure 1a–c. It is known that moisture is one of the main factors that can affect the AC breakdown voltage of dielectric insulating fluids [1]. It is expected that there are no significant changes on the relative permittivities of all of the RBDPO and CO, as shown in Figure 3a–c, which is also in line with previous studies [3,30,31]. The slight increment trends of dielectric dissipation factors of all of the RBDPO, as shown in Figure 2a–c, are also in line with previous studies [3,30,31,33]. For CO, decreasing trends of dielectric dissipation factor are found, which can be seen in Figure 2a–c. At the end of the ageing time, most of the RBDPO showed slight reductions of resistivities, as shown in Figure 4a–c. The resistivity of CO showed a clear increment trend, especially at ageing temperatures of 110 °C and 130 °C. Further studies will be needed in order to determine whether the differences on the initial moisture content and content of saturated and unsaturated fats in RBDPO and CO have any effect, especially on the dielectric dissipation factor and resistivity. Since one of the main byproducts of natural ester hydrolysis is fatty acids [39,70], it is anticipated that the acidities of all of the RBDPO and CO will increase with the increment of ageing temperature. However, as shown in Figure 8, the acidities of all of the RBDPO and CO remained low, even at an ageing temperature of 130 °C. Since the ageing of all of the RBDPO and CO were carried out under sealed conditions, it would take a longer ageing time to observe any increment in acidity. It is known that a fast and high increment of acidity for natural ester could be observed if the ageing was carried out under open conditions or in the presence of oxygen [56,71]. The effect of the natural ester hydrolysis mechanism is also not apparent on the viscosities of all of the RBDPO and CO, where the results in Figure 5 are consistent with previous studies [33,72]. This is expected, since this study was conducted under minimum contact with oxygen, which limits the oxidation processes of all of the RBDPO and CO. The oxidation of natural ester can initiate chain scission and cross-linking, which in turn lead to the changes in the molecular weight and subsequently increase the viscosity [33]. Both water scavenging and hydrolytic mechanisms are among the possible reasons why the TI of the paper for all of the RBDPO and CO maintained higher than 50% retention of TI at the end of the ageing time. Similar findings have been observed by previous studies of natural ester where the degradation rate has been slowed down by the removal and consumption of moisture via water scavenging and hydrolytic mechanisms [38,39,55]. Furthermore, the fatty acids produced as a result of the hydrolysis mechanism of natural ester can react with cellulose via transesterification, which can restrain the ageing of the paper [39]. It was also found that the vitamin E and A contents in all of the RBDPO had no effect on the trends of their dielectric and physicochemical properties throughout the ageing time, as shown in Figure 1, Figure 2, Figure 3, Figure 4, Figure 5, Figure 6, Figure 7 and Figure 8. Overall, for the practical application of RBDPO and CO in transformers, further study is required, especially on their long-term ageing under different conditions, and in the presence of copper and steel.

## 5. Conclusions

The AC breakdown voltages of all of the RBDPO and CO were not significantly affected under the current arrangement of ageing conditions. There were slight increments of dielectric dissipation factors for all of the RBDPO throughout the ageing time. On the other hand, the dielectric dissipation factor of CO decreased as the ageing progressed. The ageing had no effect on the relative permittivities of all of the samples, while there were slight reductions trends of resistivities for a few of the RBDPO. The resistivity of CO slightly increased throughout the ageing time.

All of the RBDPO and CO showed reduction trends of moisture in oil, especially at ageing temperatures of 110 °C and 130 °C. The acidities of RBDPO and CO maintained at very low values, and viscosities remained almost unchanged throughout the ageing time.

The papers aged in RBDPO and CO retained higher than 50% of the initial TI at the end of the ageing time, even at an ageing temperature of 130 °C. Based on the Weidmann model, it was shown that the paper ageing rates of RBDPO and CO significantly increased at an ageing temperature of 130 °C, as compared to 90 °C and 110 °C.

## Figures and Tables

**Table 1:** Fatty acid composition, fat, and vitamin E/A content of all of the samples.

Types of Fats	Types of Fatty Acids	RBDPOA		RBDPOB		RBDPOC		CO	
		GC (%)	MD (g)	GC (%)	MD (g)	GC (%)	MD (g)	GC (%)	MD (g)
Saturated	C6: Caproic	-	45.4	-	44.4	-	43.0	-	92.8
	C8: Caprylic	-		-		-		-	
	C10: Capric	-		-		-		-	
	C12: Lauric	0.3		0.1		0.3		48.6	
	C14: Myristic	1.1		0.9		0.9		20.0	
	C16: Palmitic	37.7		39.3		39.3		9.5	
	C18: Stearic	3.9		4.2		4.2		3.2	
Monounsaturated	C18: Oleic	42.3	43.0	41.1	43.3	43.0	43.0	6.0	3.6
Polyunsaturated	C18: Linoleic	12.4	11.6	12.2	12.2	10.4	14.0	1.1	3.6
	C18: Linolenic	0.3		0.3		0.2		-	
Vitamin E		-	4.4 × 10^−3^	-	50 × 10^−3^	-	75 × 10^−3^	-	-
Vitamin A		-	264 × 10^−6^	-	-	-	-	-	-

* CO = coconut oil, GC = Gas chromatography, MD = Manufacturer’s datasheet, RBDPO = refined, bleached, and deodorized palm oil.

**Table 2:** Ageing rates of paper for all of the samples, based on the Weidmann model.

Temperature	Samples	C_TI_ (Days^−1^)	*R*^2^	95% Confidence Bounds	
				Lower	Upper
90 °C	RBDPOA	0.597 × 10^−2^	0.611	0.199 × 10^−2^	0.995 × 10^−2^
	RBDPOB	0.598 × 10^−2^	0.487	0.137 × 10^−2^	1.059 × 10^−2^
	RBDPOC	0.6 × 10^−2^	0.747	0.071 × 10^−2^	1.129 × 10^−2^
	CO	0.523 × 10^−2^	0.439	0.107 × 10^−2^	0.94 × 10^−2^
110 °C	RBDPOA	0.6 × 10^−2^	0.72	0.109 × 10^−2^	1.091 × 10^−2^
	RBDPOB	0.388 × 10^−2^	0.877	0.183 × 10^−2^	0.594 × 10^−2^
	RBDPOC	0.415 × 10^−2^	0.825	0.129 × 10^−2^	0.702 × 10^−2^
	CO	0.492 × 10^−2^	0.928	0.305 × 10^−2^	0.68 × 10^−2^
130 °C	RBDPOA	2.504 × 10^−2^	0.814	1.058 × 10^−2^	3.949 × 10^−2^
	RBDPOB	1.877 × 10^−2^	0.694	0.552 × 10^−2^	3.201 × 10^−2^
	RBDPOC	2.805 × 10^−2^	0.424	−3.308 × 10^−5^	5.613 × 10^−2^
	CO	2.844 × 10^−2^	0.497	0.188 × 10^−2^	5.5 × 10^−2^
